# Health related quality of life and its association with social support among people living with HIV/AIDS receiving antiretroviral therapy in Ethiopia: a systematic review and meta-analysis

**DOI:** 10.1186/s12955-022-01985-z

**Published:** 2022-05-08

**Authors:** Nebiyu Mengistu, Habtamu Endashaw Hareru, Seid Shumye, Solomon Yimer, Daniel Sisay, Abdene Weya Kaso, Temesgen Muche, Chalachew Kassaw, Negasa Eshete Soboksa, Wondwosen Molla, Alemayehu Molla, Bereket Duko

**Affiliations:** 1 Department of Psychiatry, Dilla University, P.O. Box (DU): 419, Dilla, Ethiopia; 2 School of Public Health, College of Medicine and Health Science, Dilla University, P.O. Box (DU): 419, Dilla, Ethiopia; 3 Department of Human Nutrition, College of Medicine and Health Science, Dilla University, Dilla, Ethiopia; 4 Department of Midwifery, Dilla University, P.O. Box (DU): 419, Dilla, Ethiopia; 5 Curtin School of Population Health, Curtin University, Perth, WA Australia

**Keywords:** Quality of life, HIV/AIDS, Antiretroviral therapy, Social support, Ethiopia

## Abstract

**Background:**

People living with HIV/AIDS (PLWHA) are frequently confronted with severe social issues such as rejection, abandonment, criticism, and stigma. This would negatively affect their quality of life. Several studies have been conducted so far to assess factors affecting the health-related quality of life among people living with HIV/AIDS who are on antiretroviral therapy (ART) in Ethiopia. However, to our knowledge, there is no previous study that has summarized the results of the studies that investigated health-related quality of life (HRQOL) among PLWHA in Ethiopia. Therefore, the purpose of this review was to estimate the pooled prevalence of HRQOL and its association with social support among people living with HIV/AIDS (PLWHA) on ART in Ethiopia.

**Methods:**

A systematic search was carried out using several electronic databases (PubMed, Science Direct, Web of Science, and Cochrane electronic), Google Scholar, Google, and a manual search of the literature on health-related quality of life among people living with HIV/AIDS who are on ART. A Microsoft Excel data extraction sheet was used to extract pertinent data from an individual study. To assess the heterogeneity of primary articles, the Cochrane Q test statistics and the I2 test were carried out, and a random effects meta-analysis was used to estimate the pooled prevalence of HRQOL.

**Result:**

Out of the 493 articles reviewed, ten with a total of 3257 study participants were eligible for meta-analysis. The pooled prevalence of HRQOL among people living with HIV/AIDS who are on antiretroviral therapy in Ethiopia was 45.27%. We found that strong perceived social support was significantly associated with higher levels of subjectively perceived HRQOL. PLWHA who were on ART and had good social support were four times more likely to report higher HRQOL when compared to their counterparts [AOR = 4.01, 95% CI 3.07–5.23].

**Conclusion:**

A substantial number of PLWHA had poor HRQOL in Ethiopia. Social support was significantly associated with HRQOL among people living with HIV/AIDS. Hence, it’s recommended to encourage suitable intervention at every follow-up visit, and psycho-social support is also warranted to improve the quality of life.

## Introduction

According to a World Health Organization (WHO) report, there were 37.7 million people living with HIV in 2020 [1]. More than two-thirds of those with HIV/AIDS are estimated to live in Africa, and it is becoming a major public health problem, with more than 34.7 million people dying of the disease globally [1]. The impact of HIV/AIDS is more devastating in Sub-Saharan African countries. In 2017, there were 19.6 million people living with HIV/AIDS, with 53% in Eastern and Southern Africa and 6.1% in Western and Central Africa, respectively [2]. Similarly, HIV is becoming a major community concern in Ethiopia with an estimated prevalence of 1.16% among the adult population. It has been estimated that 79% of people infected with HIV know their status, of which nearly 96–99% have started receiving ART treatment [3]. By the end of 2020, 27.4 million people living with HIV have received antiretroviral therapy (ART), resulting in a global ART coverage of 73%. As a result, more efforts are required to expand treatment [1].

The United Nations Programme On HIV/AIDS (UNAIDS) has set specific targets for the identification and treatment of the disease, specifically 90-90-90, that is, HIV treatment targets of 90% of HIV patients knowing their status, 90% of those receiving ART, and 90% of those on ART experiencing viral suppression [4]. However, little attention has been given to patients' psychological, social, and financial needs [5].

Apart from the disease's biological and physical effects, many people living with HIV/AIDS endure severe social problems such as rejection, abandonment, criticism, and stigma, all of which have a poor impact on their own quality of life [6]. According to the WHO, quality of life is defined as an individual's perception of their position in life in relation to their goals, expectations, standards, and concerns in the context of the culture and value systems in which they live [7].

Various authors reported inconsistent findings regarding the prevalence of HRQOL among people living with HIV/AIDS in different regions of Ethiopia. Therefore, the main purpose of this systematic review and meta-analysis was to estimate the pooled prevalence of HRQOL and its association with social support among people living with HIV/AIDS receiving ART in Ethiopia.

## Methods

### Identification and study selection

The Preferred Reporting Items for Systematic review and Meta-Analyses (PRISMA) guidelines were used to guide this systematic review and meta-analysis [8]. We systematically searched PubMed/MEDLINE, Science Direct, Hinari, Psych INFO, and Cochrane library to find all eligible studies. Key terms used to search the literature were “prevalence” OR “magnitude” OR “proportion” OR incidence AND “health-related quality of life” OR “HRQOL” OR “quality of life” OR “QOL” AND “human immunodeficiency virus” OR “HIV infection” OR “acquired immunodeficiency syndrome” “AIDS” AND “antiretroviral therapy” OR “ART” OR “highly active anti-retroviral therapy” “HAART” AND “social support” OR “social assistance” AND “Ethiopia.” Moreover, we searched grey literature using Google searching. We have also conducted searching on organizational websites such as the WHO website to find grey literature. For studies whose full texts were not accessible, we contacted and requested the first authors via email. The search included studies published between 2013 and 2021. This review was designed in accordance with the identified characteristics of reports on the Preferred Reporting Items for Systematic Reviews and Meta-Analyses (PRISMA) [9].

### Inclusion criteria details

Primary studies that fulfilled the following criteria were considered as eligible for this systematic review and meta-analysis.

#### Study settings

Studies conducted across different regions of the country (Ethiopia) have been considered.

#### Study design

Observational studies (cross-sectional, case–control, and cohort studies) with original data reporting the prevalence of HRQOL and its association with social support among people with HIV/AIDS were considered eligible to be included in this review.

#### Language

In this review, we included articles published in English language or have English language translation.

#### Publication status

Both published and unpublished articles were included in this review.

#### Study population

Articles conducted among adult Ethiopians (equal to or age greater than 18 years) attending ART clinics were considered.

#### Measurements

Studies reporting the prevalence of HRQOL using standardized measurement tools or questionnaires such as the Short-Form 36 (SF-36) health-related quality-of-life (HRQOL) questionnaire and the Short-Form 12 (SF-12) health-related quality-of-life (HRQOL) questionnaire or other measurement tools were included.

#### Study period

Primary studies available online from 2013 to June 30, 2021 were included in this review and meta-analysis.

### Exclusion criteria

Articles studied outside of Ethiopia, reviews, editorials, letters to editors, short communications, and commentaries were excluded from the review.

## Outcome measurements

This review measured two outcomes: the first outcome was to determine the pooled average prevalence of poor HRQOL among people living with HIV/AIDS on highly active antiretroviral therapy in Ethiopia. The second outcome was to identify the association between HRQOL and social support.

### Data extraction

Seven authors (N.M, H.E, S.S, S.Y, T.M, A.M and C.K) independently screened and extracted all necessary data using a standardized data extraction format. Any disagreements among the reviewers in study selection, validity assessment, and data extraction were resolved by discussion. The data extraction format includes the first author’s name, year of publication, study region, study period, sample size, and instruments used to measure the QOL, and the prevalence of poor QOL. For the associated factors, two-by-two tables were prepared for extracting the frequency and the adjusted odds ratio was calculated.

### Quality assessment

All authors independently assessed the quality of the original articles using the Newcastle–Ottawa Scale modified for cross-sectional studies [10]. The assessment tool consists of three main segments; the first segment assesses the methodological quality of each study; the second section inspects the comparability of the studies, and the last subdivision measures the statistical analysis and the outcome of the study article. Studies scoring 6 out of 10 scales were considered as high-quality research articles and included for analysis. Differences among the authors were solved by taking the average score of their assessment results.

### Statistical procedure

Data were extracted using a Microsoft Excel spread sheet and exported to STATA version- 14 for analysis. Heterogeneity between the included articles was checked by using Q-static and I^2^ tests [11]. The results indicated a significant heterogeneity among the included studies as evidenced by I^2^ = 97.1% and *p* ≤ 0.001. Therefore, a random effects meta-analysis model was used to estimate the Der Simonian and Laird’s pooled prevalence of poor health-related quality of life. Publication bias was also examined by performing Egger’s correlation and Begg’s regression intercept tests at a 5% significant level [12]. The results of these tests indicated that there was no publication bias as evidenced by *p* = 0.051 and 0.371 in Egger’s and Begg’s tests, respectively. Moreover, subgroup analysis was done based on the regions, publication year, and sample size to minimize the random variations between the point estimates of the primary studies. The point estimates with their 95% confidence intervals were presented using texts, table and forest plots. Pooled adjusted odds ratio (AOR) was estimated to determine the association between HRQOL and social support.

## Result

### Study selection

**Fig. 1:**
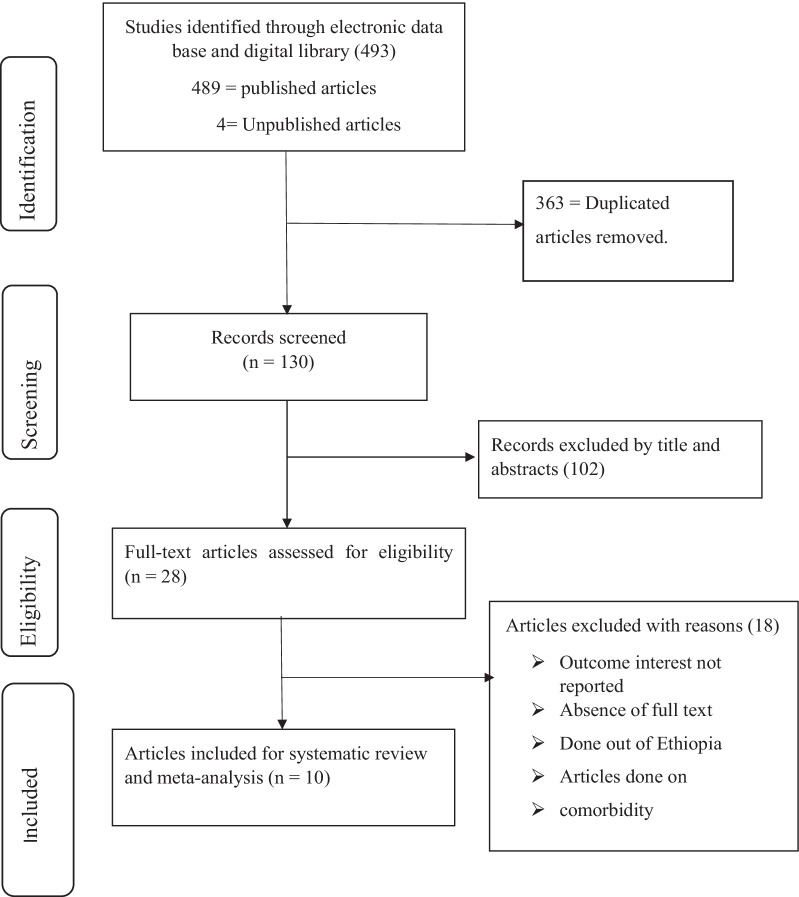
PRISMA flow diagram describes the selection of studies for a systematic review and meta-analysis of health-related quality of life of people with HIV/AIDS on ART and its association with social support for HIV/AIDS on ART in Ethiopia, 2021

A total of 493 articles were identified through electronic databases (Google Scholar, PubMed, Cochrane Library, Web of Science, and Google Science Direct) and digital library searches. After removing duplicates, a total of 130 items were retrieved, of which 102 were excluded by scanning the titles and abstracts. Twenty eight full text articles were reviewed and ten articles that met the eligibility criteria were included in the final meta-analysis (Fig. 1)*.*

### Characteristics of included studies

A total of 10 cross-sectional studies published from 2013 to 2021 were included for systematic review and meta- analysis to estimate the pooled prevalence of HRQOL. From the total studies, four studies [5, 13–15] reported the association between HRQOL and social support. Four primary studies were conducted in Amhara [16–19], four from Oromia [13, 14, 20, 21] and two from SNNPR region [5, 15] with sample size ranged 97 to 424 patients; seven studies enrolled more than 300 cases, the reviewed studies were mostly done in urban settings with the total of 3257 study participants.

The highest prevalence of poor HRQOL (58.9%) of patients with HIV/AIDS on ART was reported from a study done in the Jimma, Oromia [13] and the lowest prevalence of poor HRQOL (17.25%) was observed from the study conducted in Gondar, Amhara [18] (Table 1).

**Table 1:** Summary table for the prevalence of poor health-related quality of life of people living with HIV/AIDS on highly active antiretroviral therapy in Ethiopia included in the systematic review and meta-analysis (2021)

Authors	Region	Publication year	Study area	Sample size	Response rate	Prevalence of poor HRQOL	Assessment tool	Quality assessment
Amare A et al	Amhara	2013	Bahirdar	424	100	56.4	None	7
Ayalew MB et al	Amhara	2019	Gonder	287	100	27	WHO QOL-HIV-BREF	9
Mohammed SA et al	Amhara	2021	Dessie	251	93.62	57.4	MOS-HIV health survey	8
Surur A et al	Amhara	2017	Gondar	403	99.2	17.25	WHOQOL-HIV BREF	6
Ayeno D et al	Oromia	2020	Ambo	340	87	53	WHOQOL-HIV BREF	7
Negera Z et al	Oromia	2019	Jimma	97	97.9	58.9	WHOQOL-HIV BREF	7
Tesfaye T et al	Oromia	2018	Jimma	351	100	40.7	SF-36	8
Weldsilase YA et al	Oromia	2018	Jimma	377	91	53.5	WHOQOL-HIV BREF	8
Desta A et al	SNNPR	2020	Mizan-Tepi	311	77.2	42.9	WHOQOL-HIV BREF	6
Tesemma L et al	SNNPR	2019	ArbaMinch	416	94	47.1	WHOQOL-HIV BREF	7

### Prevalence of poor HRQOL in Ethiopia

**Fig. 2:**
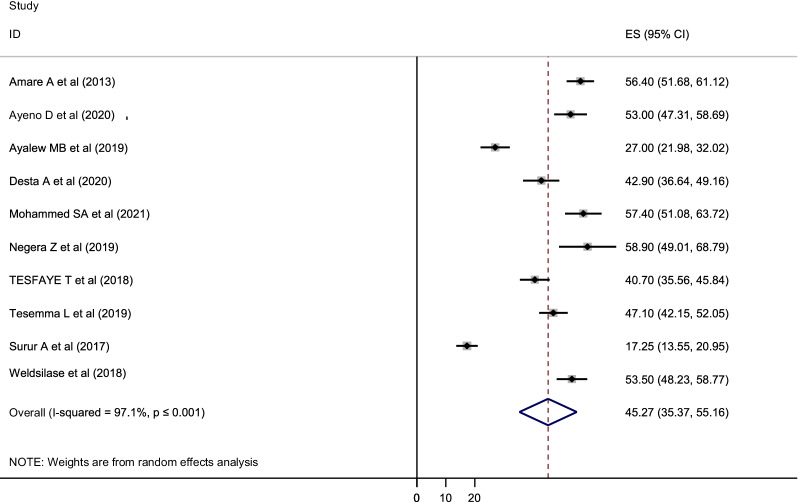
Forest plot for the pooled prevalence of poor quality of life of people living with HIV/AIDS on highly active antiretroviral therapy in Ethiopia (2021)

The pooled prevalence of HRQOL among people living with HIV/AIDS and on highly active antiretroviral therapy in Ethiopia was 45.27% with 95% CI (35.37–55.16) [5, 13–21]. A random effects model was used to estimate the pooled prevalence of poor health-related quality of life as a result of significant heterogeneity among the studies (I^2^ = 97.1%, *p* value ≤ 0.001) (Fig. 2).

### Subgroup analysis

In this meta-analysis, we performed a subgroup analysis based on the regions where the studies were conducted, publication years, and sample size.

From the three regions, the lowest prevalence [39.45% (95% CI 18.65–60.24)] of poor health-related quality of life was found in Amhara region [16–19], whereas, the highest prevalence [51.01%, (95% CI 43.43–58.60)] of poor health related quality of life was observed in Oromia region [13, 14, 20, 21].

Regarding the publication year, the prevalence of poor health-related quality of life was higher [48.32% (39.78–56.86)] among studies done after 2018 [38.08% (14.08–62.08)]. Moreover, the highest prevalence of poor health-related quality of life (46.28%) was observed among the studies (Table 2).

**Table 2:** Subgroup analysis of the prevalence of poor quality of life among peoples living with HIV/AIDS on highly active antiretroviral therapy in Ethiopia (2021) (n = 10)

Variables	Characteristics	Included studies	Prevalence rate [95% CI]
Region	Amhara	4	39.45 [18.64–60.24]
	Oromia	4	51.01 [43.42–58.60]
	SNNPR	2	45.45 [41.44–49.47]
Publication year	≤ 2018	2	38.08 [14.08–62.08]
	> 2018	8	48.32 [39.78–56.86]
Sample size	< 326	4	46.28 [30.72–61.87]
	> 326	6	44.60 [30.87–58.34]
Overall		10	45.27 [35.37–55.16]

### Sensitivity analysis

**Fig. 3:**
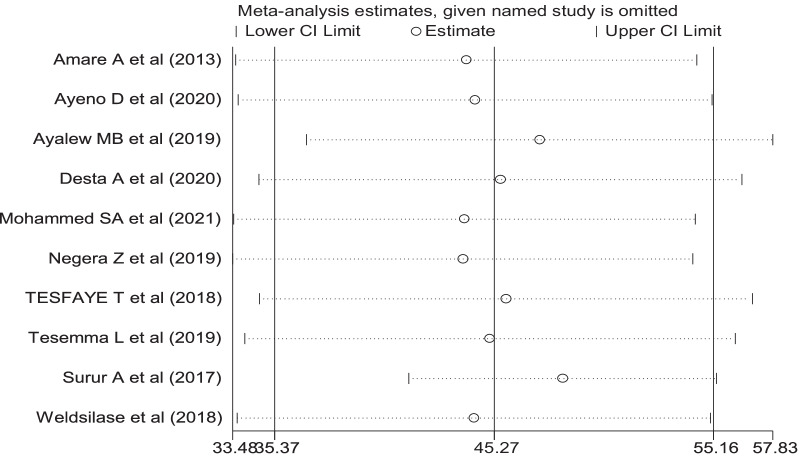
Sensitivity analysis showing the pooled proportion when the studies were omitted step by step

To investigate the influence of a single study on the overall meta-analysis, a sensitivity analysis was performed. The analysis revealed that almost all studies found within the confidence interval of the pooled prevalence, implying that they had a nearly equal effect on the total prevalence (Fig. 3).

### The association between HRQOL and social support

**Fig. 4:**
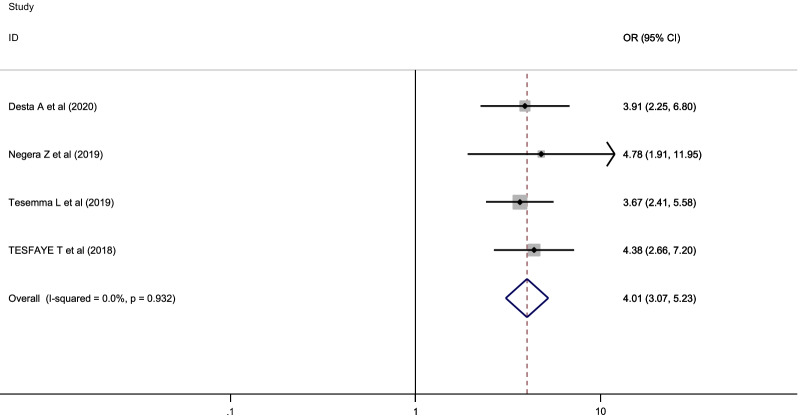
The pooled odds ratio of the association between HRQOL and social support of patients with HIV/AIDS on ART in Ethiopia, 2021

The association between HRQOL and social support was determined based on the results of four studies [5, 13–15]. The results indicated that patients with HIV/AIDS on ART who had strong social support were four times more likely to have good HRQOL than those who had poor social support [OR = 4.01, 95% CI 3.07–5.23]. After checking the fixed effect model, no change was obtained in the pooled estimate. Therefore, we had considered a random effect model to estimate the associations (I^2^ = 0.00%, *p* value = 0.932) (Fig. 4).

To identify the presence of publication bias, both Begg and Egger’s test was conducted. The results showed the absence of publication bias, evidenced with *p* values of 0.734 and 0.270, respectively.

## Discussion

In this systematic review and meta-analysis, ten studies with a total of 3257 participants were included to pool the summarized evidence on the pooled prevalence of HRQOL and its association with social support among PLWHA who are on ART in Ethiopia.

The current systematic review and meta-analysis estimated the pooled prevalence of HRQOL among PLWHA who are on ART in Ethiopia was 45.27% (95% CI 35.37–55.16). This finding is consistent with another study conducted in Iran that reported the overall mean QOL of PLWHA who are on ART was 39.13% [22] and a study from Ghana (38.3%) [23]. However, this review pooled estimate is higher than the estimate reported by studies conducted in western Africa Cameron (28.6%) [24], Nigeria (33.4%) [25] and Ghana (11.39%) [26]. This discrepancy might be observed due to variability in socio-cultural aspects, clinical status of the participants, study design, and better patient care in previous studies. As a result, updating public health policy with particular outcome modifications and support interventions should be implemented to improve HRQOL of PLWHA.

Moreover, to enhance HRQOL of people with HIV/AIDS on ART, crucial interference must be mandatory to maximize ART adherence.

We found that more perceived social support was significantly associated with higher levels of subjectively perceived HRQOL. For instance, PLWHA who are on ART and had good social support were four times more likely to report higher HRQOL when compared to their counter parts [AOR = 4.01, 95% CI 3.07–5.23]. This finding was consistent with the result of a study conducted in China [27]. Furthermore, good levels of QOL cores were also positively associated with family support. This is also in agreement with the results of a study that examined the Trajectories of Health-Related Quality of Life and Perceived Social Support Among People Living with HIV Undergoing ART [28]. The finding may be due to social support that can enhance patients adherence to the medication [29, 30] and associated with enhanced HRQOL [31]. This is also evidenced by another systematic review conducted in Low- and Middle-Income countries that suggested patients with social support were two times more likely to report good adherence to the treatment [32]. However, it is inconsistent with another systematic review and meta-analysis [33]. This may be due to some social relationships that may predispose to unhealthy behaviour’s, such as drinking alcohol, smoking, and drug use among adolescents [32].

### Limitations of the study

There are several limitations to this systematic review and meta-analysis. Firstly, all articles considered for this study were cross-sectional with small sample sizes, which could affect the pooled estimate. Secondly, the studies reviewed were only from three regions of Ethiopia, which may underrepresent the rest of the country. Furthermore, most studies conducted around the world placed a greater emphasis on HRQOL's long-term outcomes, making further discussion difficult.

## Conclusion

According to the findings of this study, a considerable number of PLWHA in Ethiopia who are on antiretroviral therapy have poor HRQOL. HRQOL was positively associated with a fourfold increase in social support. As a result, it is recommended that appropriate interventions be encouraged at all follow-up periods, as well as psychosocial support, to improve the quality of life of PLWHA.

## Data Availability

Data will be made available by request.

## Abbreviations

ART: Antiretroviral therapy
WHO: World Health Organization
HAART: Highly active
HRQOL: Health related quality of life
UNAIDS: United Nations Programme on HIV/AIDS
PLWHA: People living with HIV/AIDS

## References

[1] World Health Organization. Global progress report on HIV, viral hepatitis and sexually transmitted infections, 2021: accountability for the global health sector strategies 2016–2021: actions for impact: web annex 2: data methods. 2021.

[2] Suleiman BA, Yahaya M, Olaniyan F, Sule A, Sufiyan M (2020). Determinants of health-related quality of life among human immunodeficiency virus positive (HIV-positive) patients at Ahmadu Bello University teaching hospital, Zaria, Nigeria-2015. BMC Public Health.

[3] Girum T, Wasie A, Worku A (2018). Trend of HIV/AIDS for the last 26 years and predicting achievement of the 90-90-90 HIV prevention targets by 2020 in Ethiopia: a time series analysis. BMC Infect Dis.

[4] Algaralleh A, Altwalbeh D, Al-Tarawneh F (2020). Health-related quality of life among persons living with HIV/AIDS in Jordan: an exploratory study. HIV/AIDS (Auckland, NZ).

[5] Tesemma AL, Abate MG, Abebo ZH, Madebo WE (2019). Determinants of poor quality of life among adults living with HIV and enrolled in highly active anti-retroviral therapy at public health facilities of Arba Minch Town Administration in Southern Ethiopia. HIV/AIDS (Auckland, NZ).

[6] Vu GT, Tran BX, Hoang CL, Hall BJ, Phan HT, Ha GH (2020). Global research on quality of life of patients with HIV/AIDS: is it socio-culturally addressed?(GAPRESEARCH). Int J Environ Res Public Health.

[7] Group W (1995). The World Health Organization quality of life assessment (WHOQOL): position paper from the World Health Organization. Soc Sci Med.

[8] Liberati A, Altman DG, Tetzlaff J, Mulrow C, Gøtzsche PC, Ioannidis JP (2009). The PRISMA statement for reporting systematic reviews and meta-analyses of studies that evaluate health care interventions: explanation and elaboration. J Clin Epidemiol.

[9] Moher D, Liberati A, Tetzlaff J, Altman DG, Group T. Linee guida per il reporting di revisioni sistematiche e meta-analisi: il PRISMA Statement. PLoS Med. 2009;6(7):e1000097.

[10] Peterson J, Welch V, Losos M, Tugwell P (2011). The Newcastle–Ottawa scale (NOS) for assessing the quality of nonrandomised studies in meta-analyses.

[11] Rücker G, Schwarzer G, Carpenter JR, Schumacher M (2008). Undue reliance on I 2 in assessing heterogeneity may mislead. BMC Med Res Methodol.

[12] Egger M, Smith GD, Schneider M, Minder C (1997). Bias in meta-analysis detected by a simple, graphical test. BMJ.

[13] Zeleke Negera G, Ayele Mega T (2019). Health-related quality of life among admitted HIV/AIDS patients in selected Ethiopian tertiary care settings: a cross-sectional study. Open Public Health J..

[14] Tesfaye T, Darega J, Belachew T, Abera A (2018). Health-related quality of life and associated factors among people living with HIV/AIDS following ART clinic in Jimma University Specialized Hospital, Southwest Ethiopia: a facility-based cross-sectional study. Open Public Health J.

[15] Desta A, Biru TT, Kefale AT (2020). Health related quality of life of people receiving highly active antiretroviral therapy in Southwest Ethiopia. PLoS ONE.

[16] Alemu A, Yenealem A, Feleke A, Meseret S (2013). Health related quality of life assessment and associated factors among people on highly active antiretroviral therapy at Felege Hiwot referral hospital, Bahir Dar, north West Ethiopia. J AIDS Clin Res.

[17] Ayalew MB, Abdela OA, Solomon A, Adugna D, Siraj N, Yesuf JS (2018). Quality of life of HIV patients taking antiretroviral treatment in a resource limited setting: a case of University of Gondar comprehensive specialized hospital, Ethiopia. J Basic Clin Pharm..

[18] Surur AS, Teni FS, Wale W, Ayalew Y, Tesfaye B (2017). Health related quality of life of HIV/AIDS patients on highly active anti-retroviral therapy at a university referral hospital in Ethiopia. BMC Health Serv Res.

[19] Mohammed SA, Yitafr MG, Workneh BD, Hailu AD (2021). Health-related quality of life and associated factors among people living with human immunodeficiency virus on highly active antiretroviral therapy in North East Ethiopia: cross-sectional study. PLoS ONE.

[20] Ayeno HD, Atomsa KM, Taye GM (2020). Assessment of health-related quality of life and associated factors among HIV/AIDS patients on highly active antiretroviral therapy (HAART) at Ambo General Hospital, West Shewa, Ethiopia. HIV/AIDS (Auckland, NZ).

[21] Abebe Weldsilase Y, Likka MH, Wakayo T, Gerbaba M (2018). Health-related quality of life and associated factors among women on antiretroviral therapy in health facilities of Jimma Town, Southwest Ethiopia. Adv Public Health..

[22] Maleki MR, Derakhshani N, Azami-Aghdash S, Naderi M, Nikoomanesh M (2020). Quality of life of people with HIV/AIDS in Iran: a systematic review and meta-analysis. Iran J Public Health.

[23] Sena CE (2019). Determinants of health related quality of life in HIV-positive patients on antiretroviral therapy.

[24] Busi AN, Nsoh M, Otieno MO, Ndeso SA, Halle-Ekane GE (2021). Evaluation of quality of life and associated factors among HIV patients on antiretroviral therapy in North West region of Cameroon. Afr Health Sci.

[25] Biambo AA, Adibe MO, Liman HM, Ukwe CV (2018). Health-related quality of life of HIV-infected patients taking different antiretroviral regimens at a tertiary healthcare facility in northern Nigeria. Trop J Pharm Res.

[26] Osei-Yeboah J, Owiredu WK, Norgbe GK, Lokpo SY, Obirikorang C, Alote-Allotey E (2017). Quality of life of people living with HIV/AIDS in the Ho Municipality, Ghana: a cross-sectional study. AIDS Res Treat.

[27] Xu J-F, Ming Z-Q, Zhang Y-Q, Wang P-C, Jing J, Cheng F (2017). Family support, discrimination, and quality of life among ART-treated HIV-infected patients: a two-year study in China. Infect Dis Poverty.

[28] Gruszczyńska E, Rzeszutek M (2019). Trajectories of health-related quality of life and perceived social support among people living with HIV undergoing antiretroviral treatment: does gender matter?. Front Psychol.

[29] DiMatteo MR (2004). Social support and patient adherence to medical treatment: a meta-analysis. Health Psychol.

[30] Vagiri RV, Meyer JC, Godman B, Gous AGS (2018). Relationship between adherence and health-related quality of life among HIV-patients in South Africa: findings and implications. J AIDS HIV Res.

[31] Mannheimer SB, Matts J, Telzak E, Chesney M, Child C, Wu A (2005). Quality of life in HIV-infected individuals receiving antiretroviral therapy is related to adherence. AIDS Care.

[32] Khamarko K, Myers JJ, World Health Organization (2013). The influence of social support on the lives of HIV-infected individuals in low-and middle-income countries.

[33] Ghiasvand H, Higgs P, Noroozi M, Ghaedamini Harouni G, Hemmat M, Ahounbar E (2020). Social and demographical determinants of quality of life in people who live with HIV/AIDS infection: evidence from a meta-analysis. Biodemography Soc Biol.

